# Health outcomes, education, healthcare delivery and quality – 3052. IL-17, IL-18, TGF-beta and GMCSF levels in patients with allergic asthma: Its relation to omalizumab treatment

**DOI:** 10.1186/1939-4551-6-S1-P221

**Published:** 2013-04-23

**Authors:** Arzu Didem Yalcin, Atil Bisgin, Reginald M Gorczynski

**Affiliations:** 1 Internal Medicine, Allergy and Immunology, Education and Research Hospital, Turkey; 2 Cancer Institue, Sweden; 3 Division of Cellular & Molecular Biology, Toronto Hospital, University Health Network, Toronto, ON, Canada

## Background

Asthma is the most common serious chronic lung disease that affects people of all ages with evidence for a growing prevalence in industrialized as well as in developing countries. Allergic asthma is considered to be a Th2-dominant chronic inflammatory disease of the lungs. Th2 cells secret a panel of cytokines with several overlapping functions including IL-4, IL-5, IL-13, and GM-CSF. Increased Th2-cytokine and IgE levels and accumulation activation of Th2 cells, eosinophils and mast cells are observed in asthmatic lungs. However, recent studies focused on cell-based mechanisms for the pathogenesis of allergic asthma.

## Methods

In this study we compare the anti-IgE treatment modality in the dynamics of immune system cytokine levels of IL-8, IL-17, TGF-beta and GSCF in asthma patients who had no other any allergic disease, newly diagnosed allergic asthma patients and healthy volunteers.

## Results

The study population consisted of 14 patients suffering of SPA, 14 newly diagnosed allergic asthma patients and 14 healthy volunteers included as controls. Plasma levels of cytokines were measured. Total and specific IgE levels of anti-IgE monoclonal antibody treated patients, serum high-sensitivity C-reactive protein levels, FEV1/FVC rates and asthma control test (ACT) were measured for the clinical follow-up.

## Conclusions

We observed that patients who had SPA whom were treated with anti-IgE monoclonal antibody presented increasing levels of IL-8, TGF-β and GCSF during the treatment period of sampling times at 4 months and 18 months. However this increase was not correlated neither with serum hsCRP levels nor FEV1/FVC rates. We also noted that the levels of these cytokines did not differ between newly diagnosed allergic asthma patients, non-treated asthma patients and control.

